# Quality Tolerance Limits’ Place in the Quality Management System and Link to the Statistical Trial Design: Case Studies and Recommendations from Early Adopters

**DOI:** 10.1007/s43441-023-00504-6

**Published:** 2023-03-27

**Authors:** Marion Wolfs, Łukasz Bojarski, Steve Young, Lynne Cesario, Marcin Makowski, Linda B. Sullivan

**Affiliations:** 1 Integrated Data Analytics and Reporting, Janssen Research and Development, Graaf Engelbertlaan 75, 4837DS Breda, Netherlands; 2 Development Operations, AstraZeneca R&D BioPharmaceuticals, ul. Postepu 14, 02-675 Warsaw, Poland; 3 CluePoints Inc, King of Prussia, PA USA; 4 Pfizer R&D, New York, NY USA; 5 Data Strategy and Management, GlaxoSmithKline GmbH & Co. KG, Prinzregentenpl. 9, 81675 Munich, Germany; 6 Metrics & Performance Management, WCG, Indianapolis, IN USA

**Keywords:** Quality tolerance limits, Key risk indicators, Risk based quality management, Statistical design, Case studies, Threshold

## Abstract

Since the release of ICH E6(R2), multiple efforts have been made to interpret the requirements and suggest ways of implementing quality tolerance limits (QTLs) alongside existing risk-based quality management methodologies. While these efforts have contributed positively to developing a common understanding of QTLs, some uncertainty remains regarding implementable approaches. In this article, we review the approaches taken by some leading biopharmaceutical companies, offering recommendations for how to make QTLs most effective, what makes them ineffective, and several case studies to illustrate these concepts. This includes how best to choose QTL parameters and thresholds for a given study, how to differentiate QTLs from key risk indicators, and how QTLs relate to critical-to-quality factors and the statistical design of the trials.

## Introduction

Quality tolerance limits (QTLs) were introduced into Good Clinical Practice guidelines in 2016 with the release of ICH E6(R2) [1]. Section 5.0 of the updated ICH GCP guideline presents a set of recommendations covering a risk-based approach to managing quality in clinical trials, and QTLs are described in the following subsections:

**5.0.4:** Predefined quality tolerance limits should be established, taking into consideration the medical and statistical characteristics of the variables as well as the statistical design of the trial, to identify the systematic issues that can impact subject safety or reliability of trial results. Detection of deviations from the predefined quality tolerance limits should trigger an evaluation to determine if action is needed.

**5.0.7:** The sponsor should describe the quality management approach implemented in the trial and summarize important deviations from the predefined quality tolerance limits and remedial actions taken in the clinical study report (ICH E3, Section 9.6 Data Quality Assurance).

Several years earlier in 2013, the European Medicines Agency (EMA) published a reflection paper on risk- based quality management which also encouraged the use of QTLs to support effective monitoring of quality during clinical trials [2].

Since the release of ICH E6(R2), multiple efforts have been made to interpret the requirements and suggest ways of implementing QTLs alongside the existing risk-based quality management (RBQM) methodologies. The following publications are of particular note:

- Quality Tolerance Limits: Framework for Successful Implementation in Clinical Development (published in TIRS by in 2020) [3].
- Historical Benchmarks for Quality Tolerance Limits Parameters in Clinical Trials (published in TIRS in 2021) [4].
- Defining Quality Tolerance Limits and Key Risk Indicators that Detect Risks in a Timely Manner: Reflections from Early Adopters on Emerging Best Practices (published in Applied Clinical Trials in April 2022) [5].

While these efforts have contributed positively to developing a common understanding of QTLs, some uncertainty remains regarding implementable approaches. This includes how best to choose QTL parameters and thresholds for a given study, how to differentiate QTLs from key risk indicators (KRIs), and how QTLs relate to critical-to-quality (CtQ) factors. In this article, we review the approaches taken by some leading biopharmaceutical companies, offering recommendations for how to make QTLs most effective, what makes them ineffective, and several case studies to illustrate these concepts.

## Relationship Between QTLs and KRIs

During the conduct of a clinical trial, the quality of both the data and the processes for its collection can be monitored by several complementary quality oversight methods. This includes review of KRIs and other quality metrics, statistical data monitoring, data management and medical reviews, site monitoring, etc. it also now includes review of QTLs.

QTLs are conceptually quite similar to KRIs. Both are understood to be “risk indicators,” i.e., metrics designed to enable detection of pre-identified study risks of interest. Both involve the establishment of acceptable ranges of behavior, defined by boundary values that represent undesired or at-risk scenarios when exceeded. These boundary values are commonly referred to as “risk alert thresholds” for KRIs and “quality tolerance limits” for QTLs. Since KRIs are not mentioned in regulatory guidance, any distinctions between the two concepts can only be gleaned by comparing the conventional use of KRIs with the brief description of QTLs offered in ICH E6(R2).

- KRIs are being used to assess risk at various levels within a study—most commonly at the site level but also by region (e.g., country), by patient, and study-wide. QTLs on the other hand are understood to be focused exclusively on study-level risks, based on the ICH E6(R2) description that QTLs should “identify systematic issues that can impact subject safety or reliability of trial results” [1].
- The same ICH E6(R2) description suggests a focus for QTLs on the most critical study-level risks, i.e., “issues that can impact subject safety or reliability of trial results” [2]. KRIs are also being used to monitor important risks within a study, including those that may have a less direct and/or severe impact on subject safety or reliability of trial results.
- ICH E6(R2) establishes an expectation for QTLs to “summarize important deviations from the predefined quality tolerance limits and remedial actions taken in the clinical study report” [1]. No such expectation exists for KRIs.

We can then understand QTLs to be a designated subset of KRIs, focused on the most critical study-level risks identified for a given clinical trial. The fact that important QTL deviations should be reported in the clinical study report (CSR) further emphasizes the unique place of QTLs in the quality management system and implies that QTL parameters should refer to most critical aspects of the trial. This concept largely aligns with the comparison of KRIs and QTLs offered in both the TransCelerate BioPharma QTL paper [3] and the WCG Metrics Champion Consortium QTL Working Group QTL article [5].

Table 1 summarizes the proposed distinction between QTLs and KRIs across several considerations and reinforces the role of QTLs as a special subset of KRIs.

**Table 1:** Key Differences Between KRIs and QTLs

	KRIs	QTLs
Monitoring Levels	Study, Region, Site, Patient, etc	Study
Risk Criticality Level	High, Medium, Low	Highest
Summarize important deviations in CSR	No	Yes
Typical number per Study	10 to 25	1 to 5

While there is no specific required number of KRIs implemented per study, experience from the authors to-date shows that it typically falls within a range from 10 to 25. Organizations that have adopted QTLs are typically implementing from 1 to 5 per study. This aligns with the expectation that QTLs are reserved for monitoring only the highest study-level risks for each trial. Bhagat et al. further caution that selecting too many QTLs for a study “will dilute the importance of each QTL and the amount of time available to spend on controlling factors that contribute to each one” [3].

## Selection of QTL Parameters

QTLs—along with KRIs and all other quality oversight methods employed for a clinical trial—are a type of risk control. As such, the selection of QTLs should be guided by an assessment of risk for each study. ICH E8(R1) advises that such an evaluation of study risks be initiated during the design of the trial:

“The sponsor and other parties designing quality into a clinical study should identify the critical to quality factors. Having identified those factors, it is important to determine the risks that threaten their integrity and decide whether they can be accepted or should be mitigated, based on their probability, detectability and impact. Where it is decided that risks should be mitigated, the necessary control processes should be put in place and communicated, and the necessary actions taken to mitigate the risks.” [6]

Selection of QTLs should therefore be made for those critical-to-quality (CtQ) factors for which the risk to the study has been assessed as highest based on probability, detectability, and impact. The assessment of detectability should consider not just whether it is feasible to detect the risk during the execution of a study, but which quality oversight methods—if any—would be most effective at doing so. As previously mentioned, other options include site monitoring, medical and/or safety monitoring, data cleaning reviews, statistical data monitoring, KRIs, or some combination of these methods. For QTLs in particular, effective detection should take into consideration several factors:

1. Can a QTL parameter (i.e., risk indicator metric) be derived from data that will be available during the execution phase of the study and that clearly measures the risk?
2. Will the QTL parameter enable detection of the risk in a timely enough manner such that effective interventions are possible to address any confirmed issues?
3. Can meaningful tolerance limits be established that reliably identify when the parameter is outside of an acceptable range for the study?

Where identified CtQ-related risks do not meet the criteria for a QTL, study teams should be encouraged to monitor those risks in the manner they determine to be most appropriate, including the use of study-level, country, site, and patient-level KRIs. Various study scenarios may in fact preclude the effective use of QTLs for control of risks. For example, open-label extension studies (or any study which enrolls a population participating in a previous trial with the same compound and in the same indication) may have none or only a limited number of high risks related to recruitment of eligible patients for which the use of QTLs would be justified. The forced implementation of QTLs on such studies may instead result in increased monitoring complexity while bringing limited or no value.

Additional study scenarios that may be challenging for QTLs (as well as for KRIs), also pointed out by Bhagat et al [3], include the following:

- Short-duration studies for which there would not be sufficient time available to take remedial actions. This challenge is similarly relevant to risks that would occur only during the enrollment phase of a study and where enrollment is expected to be completed quickly.
- Studies with a small number of patients and/or relevant patient data on which to assess the risk, such that deviations from the QTL threshold would likely not be statistically significant and therefore result in a high degree of false signaling.

Consideration of the number of study participants is also of particular importance for “platform” clinical trials, which are becoming more abundant in the era of personalized medicine. These trials are typically managed under a single master protocol in which multiple treatments are evaluated simultaneously in smaller patient cohorts [7]. Good examples include “basket” studies in oncology, which are used to evaluate the drug candidate in multiple, carefully selected cohorts of patients with different types of cancer. Such cohorts may be also very heterogeneous, to an extent that precludes identification of a common, study-level, cross-cohort QTL. Even, if possible, to define, such a QTL may have a limited value from the perspective of the statistical analysis plan of such trials, as each cohort is often treated as a sub-study with cohort-specific rather than study-level endpoints.

## Determination of QTL Thresholds

It may be possible to establish QTL thresholds based on reference to available historical data from similar studies (e.g., same therapeutic area, indication, etc.), and a number of organizations are taking this approach for at least some of their QTLs. Expert knowledge provided by experienced members of the research team (sometimes referred to as “profound knowledge”) can also help to inform QTL thresholds. However, unlike manufacturing settings for which the same process and instrumentation is applied repeatedly to produce outputs for which a range of expected variability can be directly measured, clinical studies generally have unique designs and attributes (e.g., study population, investigational product, treatment arms, locations, etc.) that may make it challenging to definitively establish expected levels and variability for any given trial attribute. This may be the case regardless of the amount of relevant historical data available. Thus, an inherent underlying challenge exists in knowing to what extent any proposed limits apply to a given new study. Any uncertainty will confound the ability to identify “important deviations” in the context of ICH E6(R2).

Care should therefore be exercised in the selection and application of historical data for determining QTL thresholds. The historical data should ideally come from studies in a similar disease area, patient population, and phase of development. Moreover, caution is needed to verify if any significant process changes have taken place since the historical data were collected that assumptions made in the prior studies are appropriate for the current study, and to understand definitions or exclusions of data especially for data obtained from external sources.

A more pragmatic approach to establishing QTL thresholds relates to assessing risks related to the statistical design of the study and, in general, the risk that an insufficient amount of reliable data is delivered to successfully evaluate the key endpoints. Such scenarios also increase risk to participant safety, since participants may be exposed to study treatments and procedures while no research benefit is gained from the data collected. In these situations, the statistical design of the study can directly inform the minimum levels and types of patients and patient data required to support the endpoint analyses. Deviations from these minimum levels are then more clearly presenting a risk to the study, vs. the uncertainty associated with relying solely on historical data. It is important to note that the statistical design of the study should itself be guided by reference to historical data and/or expert knowledge, but once decided and incorporated into the study design, it establishes a clear set of requirements for that study. We see this approach as well aligned with the guidance offered in ICH E6(R2) (Sect. 5.0.4), i.e., that QTLs should take into consideration “the medical and statistical characteristics of the variables as well as the statistical design of the trial.” [1].

Table 2 presents a sample list of QTL parameters that are being used commonly to assess risks related to the statistical design of studies. Practical examples of how both the specific statistical analysis plan and historical data have been used are shared in the case studies section of this paper.

**Table 2:** Sample QTLs foCused on Statistical Design of Studies

QTL Parameter	Justification
Percentage or number of study participants randomized who do not meet inclusion/ exclusion criteria	A high number of study participants not meeting the entry criteria could have a significant impact on interpretation of the primary endpoint and overall validity of the trial results. It can also put study participants at undue risk to study drug exposure if they do not meet certain inclusion/exclusion criteria
Percentage of subjects with missing or unevaluable endpoints	A high number of study participants for whom the failure to collect study endpoint data could impact analysis and interpretation of study results
Percent or number of study participants who are non-compliant with study drug administration as defined in the protocol	Compliance with study drug which is lower than a predefined value may limit the individual exposure to study treatment. High number of study participants with low compliance may impact the interpretation of the efficacy results. Higher than per protocol doses or inadequate patient exposure to study drug, active comparator or background medication could also jeopardize study participants safety
Percentage or number of randomized study participants who were incorrectly stratified	High number of study participants who were incorrectly stratified may lead to imbalances in baseline characteristics between treatment arms and introduce biases in the data and significantly affect the outcome of a trial
Percentage or number of study subjects who used a concomitant medication specified in a study protocol as a rescue medication	High number of study participants who used rescue medication may indicate potential safety issues resulting from an inadequate treatment of an underlying disease. Higher than assumed use of a rescue medication may confound study outcome and introduce bias if it observed with a higher frequency or duration in one of study arms. This could have a significant impact on interpretation of the primary endpoint and overall validity of the trial results

## QTL Case Studies

### Case Study 1: QTL Parameter Selection & rationale, QTL and KRIs Monitoring

When “Percentage/# of subjects with missing or unevaluable endpoints” has been selected as a QTL parameter, it is important to understand the type of endpoint to get to a meaningful QTL. Different type of endpoints are, for example, a quantitative measurement representing a specific measure or count, binary clinical outcome indicating whether an event has occurred, the time to occurrence of an event of interest or survival time, or a composite endpoint consists of multiple outcome measures. Assessment considerations to get to a meaningful QTL parameter related to endpoints are as follows:

- Understanding how missing data and dates and will be imputed for different types of endpoints
- Understanding how the primary endpoint will be calculated and what data are critical for the calculation
- Dates of all events contributing to the complex endpoint are important
- What other parameters are important for endpoint definition and censoring purposes

**Figure 1:**
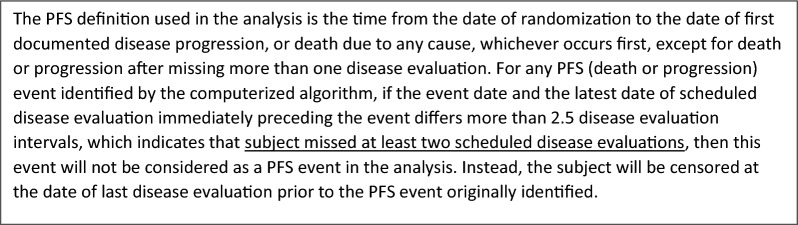
Statistical analysis plan (excerpt)

In this case study, the endpoint of three studies in a program is the time to occurrence of an event, in this case the time to progression of disease. To determine the progression of disease, serum and urine samples are collected during disease evaluation visits and analyzed. It is critical for these studies that the subjects do not miss these study visits to have the samples collected. To monitor if sites and subjects follow the process as specified in the protocol and ensure these visits are happening appropriately, a KRI is put in place to monitor the missed evaluation visits. The KRI identifies the subjects missing at least one evaluation visit in order to provide time for taking action to lower the chance of a second consecutive disease evaluation being missed. To understand the actual impact on the endpoint analysis, hence, to determine the QTL parameter, information in the statistical analysis plans is used (Fig. 1). A single missed disease evaluation or randomly missed disease evaluation will not impact the statistical analyses. Subject with 2 or more consecutive missed disease evaluation will not be considered as a PFS in the analyses, which could potentially influence the study outcome. For the three studies in the program in Table 3, you can find an overview of the QTL and KRI parameters, thresholds, and justification.

**Table 3:** Case Study 1—QTL Parameters and Thresholds for 3 Studies in a Program

Critical to Quality Factor	KRI or QTL	Parameter	Threshold Study 1	Threshold Study 2	Threshold Study 3	Definition and Justification for the QTL and KRI Parameter
Critical data to determine disease progression are collected during disease evaluation (DE) visits (collection of serum and urine samples)	QTL	% of subjects with 2 or more consecutive missing or unevaluable DE	1.1%	0.4%	0.3%	For any PFS (death or progression) event identified by the computerized algorithm, if the event date and the latest date of scheduled disease evaluation immediately preceding the event differs more than 2.5 disease evaluation intervals, which indicates that subject missed at least two scheduled disease evaluations, then this event will not be considered as a PFS event in the analysis. Instead, the subject will be censored at the date of last disease evaluation prior to the PFS event originally identified The QTL threshold has been defined based on historical data and scenario running based on study assumptions
	KRI	% of subjects with at least one missing or unevaluable DE	35%	20%	20%	The KRI enables identification of subjects and sites with a higher level than expected single missed (random) disease evaluations, which increases the chance of a consecutive disease evaluation visit being missed The KRI thresholds have been defined based on historical data and a statical methodology to determine outliers

ENDPOINT: Progression-free survival (PFS) is defined as the time from the date of randomization to the date of first documented disease progression, or death due to any cause, whichever occurs first.

CRITICIAL DATA: Critical data that are used to determine the disease progression are collected during disease evaluation visits (collection of serum and urine samples).

QTL PARAMETER: % of subjects who missed 2 or more subsequent disease evaluations.

### Case Study 2: QTL Threshold Definition Based on Historical Data, QTL and KRI Monitoring, QTL Breach, and Corrective Actions

In this case study, the clinical study team defined a QTL of 4% of randomized patients who will be at risk of unknown survival status at the time of final study analysis. The QTL parameter is related to a CtQ which is patient retention. This CtQ has been identified as relevant for this study as the overall survival is a primary efficacy measure. High number of patients with unknown survival status at the time of the final analysis may have a negative impact on interpretability of the overall survival endpoint. The threshold of 4% has been defined based on the patient retention issue rates observed in similar studies and other sponsors studies (based on ct.gov database) and a concept of a “profound knowledge” by cross-functional study team, which included medical, statistical, operational, and CM/RBQM experts. Table 4 provides an overview of the QTL and related KRI, as well as the thresholds and justifications.

**Table 4:** Case Study 2—QTL and Related KRI, as well as the Thresholds and Justifications

Critical to quality factor	KRI or QTL	Parameter and threshold	Definition and Justification
Patient retention	QTL	4% of randomized patients who are at risk of unknown survival status at the time of final study analysis	Overall survival is a primary efficacy measure – a high number of patients with unknown survival status at the time of the final analysis may have a negative impact on interpretability of the overall survival endpoint The QTL threshold of 4% has been defined based on the patient retention issue rates observed in similar Sponsor “A” studies and other sponsors’ studies (based on ct.gov database) and a concept of a “profound knowledge” by cross functional study team, which included medical, statistical, operational and CM/RBQM experts The KRI threshold has been defined to trigger CRAs action to verify patient status with site staff whenever patient at risk of unknown survival status at the time of final study analysis is detected at site. The KRI threshold has been defined to trigger CRAs action to verify patient's status with the site staff when that patient is at risk of not having a known survival status for the final study analysis
	KRI (site level)	The first and every subsequent patient randomized at site who is at risk of unknown survival status at the time of final study analysis	

**Figure 2:**
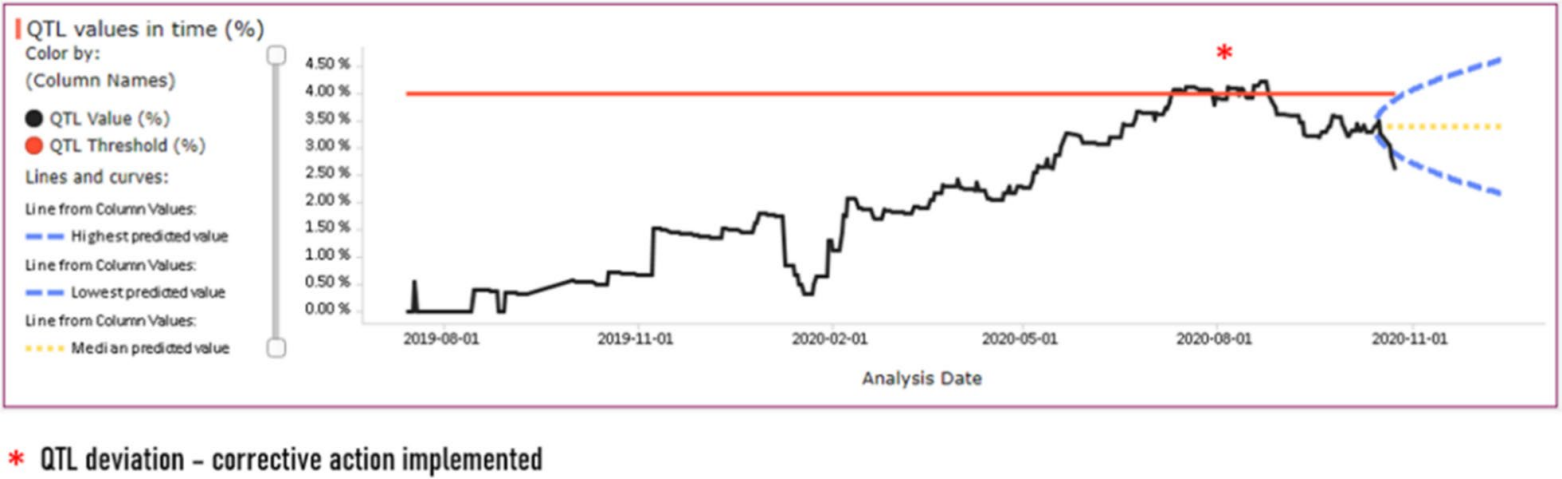
Percent of Currently Randomized Patients at Risk of Having an Unknown Survival Status at the Final Analysis in Relation to a QTL 4% Threshold for Corrective Action Initiation.

Study performance against the predefined QTL was monitored by the central monitoring system. Patients with confirmed consent withdrawals and “other termination” selections in the eCRF were included in the analysis. The QTL parameter is expressed as the percentage of patients who withdrew vs all patients randomized to date and plotted against time (Fig. 2).

The QTL analysis is coupled with an automated site-level KRI analysis, which is used to notify a site CRA whenever a new consent withdrawal is recorded in the eCRF, or whenever a patient at risk of loss to follow-up is identified at a site based on predefined criteria. In response to the notification, the CRA is expected to contact the site to confirm patient status, and to check for common issues such as documenting IP discontinuation as consent withdrawal.

Data for QTL and KRI analysis are refreshed on a daily basis and CRA notification is triggered as soon as a KRI threshold is breached. In turn, the QTL is reviewed in monthly intervals (determined as the minimum time to observe trending on a study level) and its status and/or deviations are discussed with a cross-functional study team.

The Study team evaluates the QTL in the context of site-level KRIs to identify the sites which contribute the most to unfavorable QTL trending or a QTL breach. The KRI is not evaluated in the QTL context. CRA action is required whenever KRI trigger is met irrespectively from the QTL status.

In this case, a QTL deviation was observed, which triggered a root cause analysis that indicated the following potential root causes:

1. Limited understanding at some sites that patient discontinuation from study treatment does not necessarily cause withdrawal from participation in the study and patients may consent to survival status follow-up calls.
2. Relatively high contribution of patients with “other termination” status in the analyzed sample, which may be indicative that patient status has been inappropriately reflected in the eCRF.

The team implemented the following preventive and corrective actions:

1. Re-training on consent withdrawals vs study treatment discontinuation and eCRF completion guidelines were deployed to all study sites.
2. Extensive data cleaning was applied to sites which indicated patients with “other termination” status, which led to identification of study treatment withdrawals recorded in eCRF as study terminations.

The actions taken in response to the QTL deviation turned out to be effective as indicated by the percentage of issues dropping below of the QTL threshold in the following months of observation.

### Case Study 3: QTL Parameter and Threshold Definitions, QTL and KRI Monitoring, QTL Breach, and Corrective Actions

The QTL process was introduced at this sponsor in 2020. The implementation is consistent with the TransCelerate framework described by Bhagat et al [3] and Makowski et al [4]. In short, the process of establishing and monitoring QTLs on a study level is cross-functional with biostatistics and central monitoring playing a significant role. Study teams have freedom in selecting the parameters (i.e., there is no pre-specified list); however, the parameters must relate to critical processes and data. In practice, parameters pertaining the completeness of primary endpoint data are selected in most studies. Others often pertain to eligibility- or intervention-related protocol deviations. The thresholds are determined based on historical data and statistical and medical characteristics of the trials. The recommended number of QTLs set for a study is 1–5.

The case presented here comes from a trial studying effectiveness of intervention in preventing respiratory failure in patients with an acute respiratory condition. The primary endpoint was a physician-administered scale measuring the level of respiratory support needed at day 28. The study was characterized with rapid recruitment. Prior to study start, the study team decided to implement a QTL based on a parameter measuring the completeness of primary endpoint data. The parameter was built in the following way: the numerator was the number of patients randomized more than 35 days before that had primary endpoint data entered; the denominator was all patients randomized more than 35 days before. (35 days was chosen vs. 28 days to account for both allowable visit window and data entry time). The QTL had a one-sided lower limit of 80% with secondary limit at 90% (Table 5). Less than 90% completeness of primary endpoint data, the secondary limit, was planned to trigger corrective action at the site and country levels while less than 80% completeness of primary endpoint data was planned to trigger study-level action. No additional related KRI was implemented in the study to trigger actions on site or country level, because a study-specific data visualization of the QTL was used to understand whether there were any site, country, or other correlations of data missingness.

**Table 5:** Case Study 3—QTL with Primary and Secondary Thresholds, Definitions, and Justifications

Critical to quality factor	QTL parameter	QTL threshold	Definition and justification
Primary endpoint data derived from a physician-administered scale measuring the level of respiratory support needed at day 28	Proportion of patients with the primary endpoint value entered into the eCRF to all patients randomized more than 35 days before	One-sided lower limit of 80% with secondary upper limit at 90%	The trial is studying the effectiveness of intervention in preventing respiratory failure in patients with an acute respiratory condition. Prior to the study, the study team implemented a QTL based on a parameter measuring the completeness of primary endpoint data in the CRF for all patients randomized more than 35 days before. Seven additional days beyond the 28-day assessment date was selected to account for visit window and data entry time The thresholds were set up based on analysis of historical data analysis of studies of similar design (acute indication, similar therapeutic area and endpoint)

**Fig. 3:**
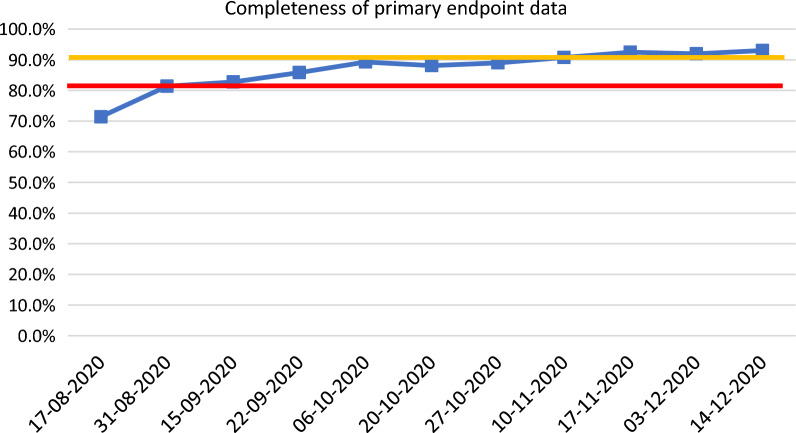
The Chart Presents the Completeness of Primary Endpoint Data During the Study. The sss) and secondary (amber) QTL limits.

During the study, the QTL was monitored by the study team. The results of monitoring are presented in Fig. 3. From the very beginning of the study, the parameter was outside of the tolerance limit. The study team performed the root cause analysis and implemented effective corrective actions both aimed at the sites where data missingness was the highest, but also study-wide addressing all sites. Over time, although the absolute number of missing primary endpoint assessments was still rising, the proportion measured by the QTL parameter returned to acceptable levels.

## Summary of QTL Recommendations

The conceptual framework and motivation for QTLs outlined in ICH E6(R2) are welcome, but flexibility is needed in the application of the concept.

The authors recommend the following:

- Selection of QTLs should be for critical-to-quality (CtQ) factors for which the risk to the study has been assessed as highest—based on probability, detectability, and impact—and for which the risk is indeed measurable (i.e., possible to be quantified during study execution).
- Where identified CtQ-related risks do not meet the criteria for a QTL, study teams should be encouraged to control those risks in the manner they determine to be most appropriate. This may include the use of study-level KRIs, clinical and safety data trending reviews, data management reviews, medical and safety monitoring, site monitoring, or other appropriate controls.
- Reference to historical data is an important consideration and may help to inform the identification of QTL thresholds. However, the applicability of a given set of historical data to a prospective study design may be questionable given the unique characteristics of each new study. Alternatively, the statistical design of trials can often be referenced to establish clear limits beyond which the interpretability of trial results may be jeopardized. We see this approach as well aligned with the guidance offered in ICH E6 (R2) (Sect. 5.0.4), i.e., that QTLs should take into consideration “the medical and statistical characteristics of the variables as well as the statistical design of the trial …”.
- QTLs may not be applicable to very short-duration studies and/or studies with small patient data volumes.
- While there is no specific required number of KRIs implemented per study, experience to-date shows that it typically falls within a range from 10 to 25. Organizations that have adopted QTLs are typically implementing from 1 to 5 per study. This aligns with the expectation that QTLs are reserved for monitoring only the highest study-level risks for each trial. Bhagat et al. further caution that selecting too many QTLs for a study “will dilute the importance of each QTL and the amount of time available to spend on controlling factors that contribute to each one.”. [3]
