# Correction: Quality Tolerance Limits’ Place in the Quality Management System and Link to the Statistical Trial Design: Case Studies and Recommendations from Early Adopters

**DOI:** 10.1007/s43441-023-00546-w

**Published:** 2023-06-10

**Authors:** Marion Wolfs, Łukasz Bojarski, Steve Young, Lynne Cesario, Marcin Makowski, Linda B. Sullivan

**Affiliations:** 1 Integrated Data Analytics and Reporting, Janssen Research and Development, Graaf Engelbertlaan 75, 4837DS Breda, Netherlands; 2 Development Operations, AstraZeneca R&D BioPharmaceuticals, ul. Postepu 14, 02-675 Warsaw, Poland; 3 CluePoints Inc, King of Prussia, PA USA; 4 Pfizer R&D, New York, NY USA; 5 Data Strategy and Management, GlaxoSmithKline GmbH & Co. KG, Prinzregentenpl. 9, 81675 Munich, Germany; 6 Metrics & Performance Management, WCG, Indianapolis, IN USA

**Correction: Therapeutic Innovation & Regulatory Science** 10.1007/s43441-023-00504-6

The article Quality Tolerance Limits’ Place in the Quality Management System and Link to the Statistical Trial Design: Case Studies and Recommendations from Early Adopters, written by Marion Wolfs, Łukasz Bojarski, Steve Young, Lynne Cesario, Marcin Makowski, and Linda B. Sullivan was originally published electronically on the publisher’s internet portal on 27 March 2023 without open access. With the author(s)’ decision to opt for Open Choice, the copyright of the article changed on 24 May 2023 to ©The Author(s) 2023, and the article is forthwith distributed under a Creative Commons Attribution CC BY 4.0.

